# Analysis of the relationship between tuberculosis-related mortality and nitrous oxide emission levels in the world with the environmental Kuznets curve method

**DOI:** 10.55730/1300-0144.5439

**Published:** 2022-06-13

**Authors:** Şerife TORUN, Kadir YILMAZ, Şevket ÖZKAYA, Şebnem YOSUNKAYA, Şule AKÇAY

**Affiliations:** 1 Department of Pulmonary Diseases, Faculty of Medicine, Konya Practice and Research Hospital, Başkent University, Konya, Turkey; 2 Department of Statistics, İstanbul Commerce University, İstanbul, Turkey; 3 Department of Pulmonary Diseases, Faculty of Medicine, Bahçeşehir University, İstanbul, Turkey; 4 Department of Pulmonary Diseases, Faculty of Medicine, Necmettin Erbakan University, Konya, Turkey; 5 Department of Pulmonary Diseases, Faculty of Medicine, Başkent University, Ankara, Turkey

**Keywords:** Tuberculosis, nitrous oxide, environmental Kuznets curve, mortality

## Abstract

**Background/aim:**

It was aimed to analyze the relationship between tuberculosis-related mortality and nitrous oxide emission levels in the world with the Environmental Kuznets Curve (EKC) Method.

**Materials and methods:**

WHO ICD-10 mortality list data and the World Bank Country Data (WBCD) were used between 1997 and 2017 for 12 countries. Cubic regression analysis was used for EKC Analysis.

**Results:**

The difference between male and female deaths between 1996 and 1998 has increased sharply since 1999. Male deaths consistently occurred significantly more than female deaths. There was a significant and negative correlation between Nitrous oxide emissions (% change from 1990) and tuberculosis-related deaths, whereas there were significant and positive correlations between Nitrous oxide emissions in the energy sector (% of total) and tuberculosis-related deaths (p < 0.01). EKC analysis results showed that there is a U shaped between tuberculosis-related mortality and nitrous oxide emission levels in the world.

**Conclusion:**

Research results show that the relationship between nitrous oxide change and mortality is negative in the short term and positive in the long term. Therefore, although nitrous oxide gases cause respiratory diseases and mortality, it may be possible to transform a harmful environmental factor into a positive by developing devices or methods that will convert these gases into free radicals.

## 1. Introduction

Environmental factors play an important role in tuberculosis. In the studies conducted in this field in the literature, the fact that the disease is more common in the working male gender is shown as evidence for this situation [1–4]. In addition, there are studies that professionally support the relationship of deaths caused by tuberculosis with environmental factors [5–8].

Nitric oxide gas is the free radical of nitrous oxide gas, which is an environmental pollutant gas. A gas does not only need to be in a laboratory environment to transform into a free radical, but other reasons can cause gas to turn into a free radical [9–11]. In some studies in the literature, positive results have been reported in treatment with nitric oxide gas in tuberculosis patients [12–14].

The Environmental Kuznets curve method (EKC) was first put forward by Kuznets in 1955 and later reconsidered in many studies, it is called a hypothesis that reveals an inverted-U-shaped relationship between various factors in environmental pollution, economy, and health-related issues, first increasing and then decreasing. It was aimed to analyze the relationship between tuberculosis-related mortality and nitrous oxide emission levels in the world with the EKC.

## 2. Materials and methods

### 2.1. Design and data collection

WHO ICD-10 mortality list data and the World Bank Country Data (WBCD) were used for data collection. In the WHO ICD-10 mortality list, two variables as respiratory tuberculosis and other tuberculosis-related death data were used with the following WHO-ICD-10 codes: 1005-A15-A16: Respiratory tuberculosis and 1006-A17-A19: Other tuberculosis. WHO has reported mortality data between 1997 and 2017 for 12 countries (Seychelles, Brunei Darussalam, Cyprus, Oman, Sri Lanka, Syrian Arab Republic, Azerbaijan, Belarus, Kazakhstan, Russian Federation, Turkmenistan, and Ukraine). WHO also published ICD-11 mortality data recently, but this data also includes as same data as ICD-10 due to pandemics and lack of data collection in 2020.

Nitrous oxide emission data were collected from WBCD with three subcategories including the agricultural, energy sector, and change parameters. Codes for variables were EN.ATM.NOXE.AG.ZS-Agricultural nitrous oxide emissions (% of total), EN.ATM.NOXE.ZG-Nitrous oxide emissions (% change from 1990), and EN.ATM.NOXE.EG.ZS-Nitrous oxide emissions in the energy sector (% of total).

### 1.2. Statistical analysis

Variables were described with means and standard deviations. Spearman’s rho correlation was used for the analysis of the relationship between research parameters. Cubic regression analysis was used for environmental Kuznets curve (EKC) Analysis. SPSS 17.0 for windows was used for analysis with a 95% confidence interval.

## 2. Results

**Figure 1:**
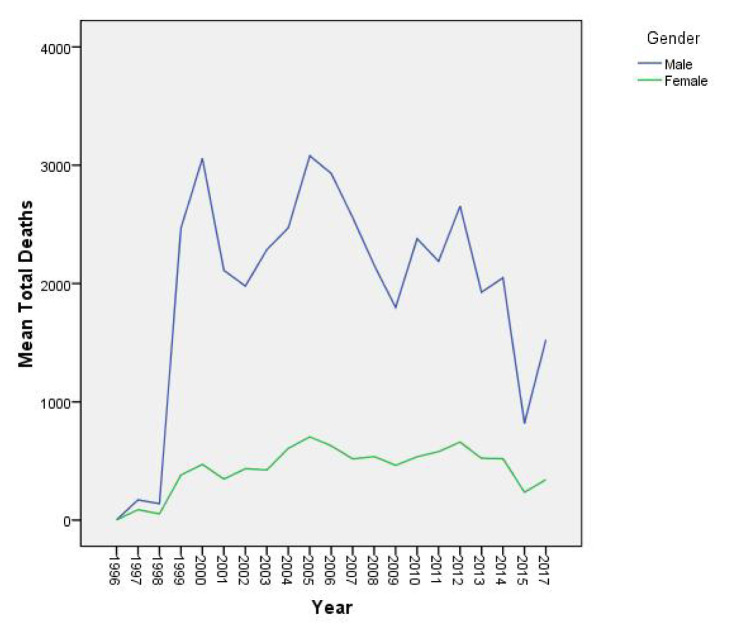
Tuberculosis related mortality based on gender and year for all countries.

The difference between male and female deaths between 1996 and 1998 has increased sharply since 1999. Male deaths consistently occurred significantly more than female deaths, although the distributions for both genders varied over time (Figure 1).

The mean tuberculosis-related death rate for all years was the highest in the Russian Federation and the lowest in Seychelles. Agricultural nitrous oxide emission mean was the Highest in Azerbaijan and the lowest in Brunei Darussalam. Nitrous oxide emissions mean in the energy sector was the highest in Seychelles, and the lowest in Turkmenistan. The increase in nitrous oxide emission was the highest in Oman (Table 1).

**Figure 2:**
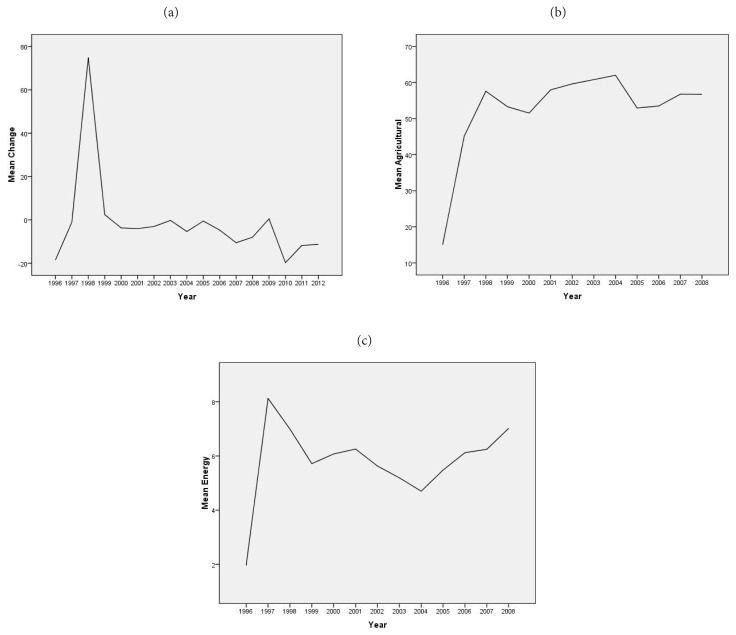
Nitrous oxide emissions changes for all countries nitrous oxide emissions (a) agricultural (b) and energy sector (c).

Spearman’s rho correlation analysis showed that there was a significant and negative correlation between nitrous oxide emissions (% change from 1990) and tuberculosis-related deaths, whereas there were significant and positive correlations between nitrous oxide emissions in the energy sector (% of total) and tuberculosis related deaths (p < 0.01). In addition, year and gender were also cofounders of tuberculosis-related total death (Table 2) (Figure 2).

In 1998, the mean change in nitrous oxide emissions was the highest for all countries and at all-time intervals. After a sharp reduction in 1999, its trend was in decreasing direction (Figure 2.a). Both agricultural nitrous oxide emissions (Figure 2.b) and energy sector nitrous oxide emissions (Figure 2.c) were the highest in 1998, and showed an unstable trend between 1999 and 2008.

Since consumption and change parameters for nitrous oxide are cointegrated parameters, the change parameter was used because of a high correlation coefficient. Cubic regression analysis model was as follows (Akyıldız, 2008:141) [15].

**Tuberculosis related deaths = β** *_0_* **+ β** *_1_* **(nitrous oxide emissions (% change from 1990)) + β** *_2_* **(nitrous oxide emissions (% change from 1990))** **^2^** **+ β** *_3_* **(nitrous oxide emissions (% change from 1990))** **^3^**

According to environmental Kuznets curve model, If:

β1 = β2 = β3, there is no relationship between variables.

β1 > 0, β2 = β3 = 0, there is a linear relationship between variables.

β1 > 0, β2 < 0 and β3 = 0, there is an “inverse U shaped” between variables, and Environmental Kuznets Curve approach is acceptable.

β1 < 0, β2 > 0 and β3 = 0, there is a “U shaped” between variables, and Environmental Kuznets Curve approach is acceptable.

β1 > 0, β2 < 0 and β3 > 0, there is an “N shaped” between variables, and Environmental Kuznets Curve approach is acceptable.

β1 < 0, β2 > 0 and β3 < 0, there is an “inverse N shaped” between variables, and Environmental Kuznets Curve approach is acceptable.

According to cubic regression analysis results, the explaining power of the model was 10.8% with a significant F value (p < 0.01). The contribution of all parameters was significant (p < 0.01). After analysis, the equation was found as follows (Table 3):

**Tuberculosis related deaths =** −29.378 × (nitrous oxide emissions (% change from 1990)) + 0.370 × (nitrous oxide emissions (% change from 1990))^2^ + 0.000 × (Nitrous oxide emissions (% change from 1990))^3^

Regression coefficients showed that β1 < 0, β2 > 0 and β3 = 0, there is a “U shaped” between variables, and Environmental Kuznet’s Curve approach is acceptable (Table 3).

## 3. Discussion

Tuberculosis is a disease whose mortality is decreasing and treatable. However, like other diseases, early diagnosis is important in tuberculosis [16–19]. For this, it is important to know risk factors and to evaluate environmental factors [20–22]. In some studies on environmental factors, it has been reported that nitric oxide has been successful in the treatment of tuberculosis [23–25]. In this study, the possibility of turning nitrous oxide gas, known as a polluting gas, into nitric oxide gas, which is a free radical, for various reasons (such as radiation, excessive heat, chemical uses, CFC gases), was evaluated. The relationship of this situation with deaths caused by tuberculosis was investigated.

Studies on deaths caused by tuberculosis report that mortality levels due to tuberculosis are higher in males than females [26–28]. In our study, it was observed that deaths due to tuberculosis were higher in males and the difference was statistically significant. In both sexes, significant and drastic changes were observed in the distribution of mortality by years between 1996 and 1998 and 2015. In 1999, a very sharp increase was seen in both men and women.

Nitrous oxide is known as a polluting gas that turns into a free radical and creates nitric oxide gas. On the other hand, various transformative factors, such as radiation, excessive heat, chemical usage, CFC gases, and similar interactions may be required for the formation of free radical gases [29–31]. The World Bank gave approval for the use of nitrous oxide gas in the agriculture and energy sectors in 1996, greatly explaining the increase in environmental associated with this gas. Although factors that can cause the conversion of this gas into free radicals in agricultural use are limited, there is a considerably higher potential for free radical formation in the energy sector. Hence, use in the energy sector is likely to have a significant relationship with the changes in tuberculosis-related mortality. In our study, the relationship between agricultural consumption and mortality was not significant. However, the relationship between industrial use (energy sector emission) and mortality was significant, showing a positive relationship. Additionally, there was a negative relationship between nitric oxide change since 1996 and mortality. In this respect, it can be stated that its harmful effects are greater than its positive effects on tuberculosis.

In a review exploring the value of nitric oxide in tuberculosis treatment, the authors conveyed ideas that the antimycobacterial effect of nitric oxide could be utilized as a direct therapy for pulmonary tuberculosis infections, in the form of inhalation [23]. This suggestion is supported by earlier [24] and recent studies [25], which have demonstrated that nitric oxide secretion supplements the antimycobacterial activity of macrophages and that *M. tuberculosis* inhibits host nitric oxide production, respectively. However, it has been stated that if nitric oxide inhalation was to be utilized, treatments should be reserved for specific patient groups without possible contraindications for nitric oxide, including those with comorbidities and various predisposing states [23]. Although it is evident that nitric oxide treatment and nitrous oxide pollution are completely different categories in this context, the conversion of nitrous oxide into nitric oxide within the lung (or prior to inhalation) could explain the relationship between mortality and nitrous oxide levels observed in our study. Of note, since nitrous oxide can cause respiratory difficulties in relation to its anesthetic effects [32], prolonged exposure or exceedingly high levels would result in negative consequences in patients with pulmonary infections such as tuberculosis.

In this research, the use of panel data analysis in the medical area is one of the positive contributions of the study. Panel data analysis allows evaluating large-scale parameters in different samples and time series [33–35]. In our research, both vertical (years) and horizontal (countries) data were effectively examined by using panel data analysis.

According to the results of EKC analysis, there was a U-shaped relationship with nitric oxide consumption. There is a change in the U-shaped relationship, which is negative at first and then positive. Therefore, increasing nitric oxide gas emission to a certain level decreases the mortality due to tuberculosis and increases mortality when it exceeds a certain level.

## 4. Conclusion

Our findings show that the relationship between nitric oxide change and tuberculosis mortality is negative in the short term and positive in the long term. Therefore, although nitrous oxide may be associated with respiratory diseases and mortality, considering studies that suggest beneficial effects of nitric oxide in the treatment of tuberculosis it may be possible to transform a harmful factor into a positive by developing devices or methods that could convert these gases into free radicals. However, it must be taken into account that such treatments should be limited to specific patients according to the results of future studies.

## Figures and Tables

**Table 1:** Tuberculosis related deaths and nitrous oxide emission rates according to countries for all years.

Country	Tuberculosis related deaths	Agricultural nitrous oxide emissions (% of total)	Nitrous oxide emissions (% change from 1990)	Nitrous oxide emissions in energy sector (% of total)
Seychelles	1.38 ± 0.87	25.65 ± 4.32	50.85 ± 23.01	**19.09** ± **3.66**
Brunei Darussalam	2.98 ± 2.02	19.64 ± 5.87	−12.56 ± 70.53	2.74 ± 1.08
Cyprus	2.00 ± 0.01	71.59 ± 0.01	17.68 ± 0.01	8.53 ± 0.01
Oman	4.33 ± 4.16		**98.31** ± **0.01**	
Sri Lanka	237.21 ± 292.11	67.49 ± 1.26	14.06 ± 4.12	12.12 ± 0.47
Syrian Arab Republic	13.98 ± 11.41	76.52 ± 2.24	26.15 ± 13.83	5.25 ± 0.66
Azerbaijan	244.31 ± 314.21	**81.20** ± **0.66**	−12.82 ± 4.59	2.86 ± 0.21
Belarus	193.19 ± 247.81	70.23 ± 1.21	−38.11 ± 4.72	5.17 ± 0.26
Kazakhstan	607.00 ± 793.43	68.18 ± 3.80	−46.23 ± 1.79	7.06 ± 0.53
Russian Federation	**6375.31** ± **7836.24**	41.73 ± 6.99	−50.43 ± 11.52	8.38 ± 2.07
Turkmenistan	160.28 ± 193.21	73.32 ± 3.46	86.16 ± 35.86	1.88 ± 0.20
Ukraine	1967.50 ± 1751.61	43.31 ± 1.76	−**57.71** ± **5.43**	5.32 ± 0.38

**Table 2:** Spearman’s rho correlation for relationship between tuberculosis related deaths and related parameters.

Total Deaths	r	p
Agricultural nitrous oxide emissions (% of total)	0.089	0.146
Nitrous oxide emissions (% change from 1990)	−0.560**	0.000
Nitrous oxide emissions in energy sector (% of total)	0.296**	0.000

** p < 0.01

**Table 3:** Cubic regression analysis results for tuberculosis related deaths and nitrous oxide emissions (% change from 1990).

		Unstandardized coefficients		Standardized coefficients	t	p
		β	Std. Error	Beta		
(Constant)- **β_0_**		293.974	319.073		0.921	0.358
(Nitrous oxide emissions (% change from 1990))- **β_1_**	−29.378	4.522	−0.423	−6.497	0.000
(Nitrous oxide emissions (% change from 1990))^2^- **β_2_**	0.370	0.093	0.770	4.000	0.000
(Nitrous oxide emissions (% change from 1990))^3^- **β_3_**	0.000	0.000	−0.559	−3.089	0.002

R^2^: 0.108; f: 15.365; p < 0.01
